# Establishment and neural differentiation of neural crest‐derived stem cells from human dental pulp in serum‐free conditions

**DOI:** 10.1002/sctm.20-0037

**Published:** 2020-07-07

**Authors:** Oscar O. Solis‐Castro, Fiona M. Boissonade, Marcelo N. Rivolta

**Affiliations:** ^1^ Centre for Stem Cell Biology, Department of Biomedical Science University of Sheffield Sheffield UK; ^2^ School of Clinical Dentistry University of Sheffield Sheffield UK; ^3^ The Neuroscience Institute University of Sheffield Sheffield UK

**Keywords:** autologous, cellular therapy, clinical translation, neural crest‐derived stem cells, neural induction, serum‐free media, tissue‐specific stem cells

## Abstract

The potential of obtaining cell cultures with neural crest resemblance (neural crest‐derived stem cells [NCSCs]) from dental‐related tissues, including human dental pulp cells (hDPCs), has been discussed in the literature. However, most reports include the use of serum‐rich conditions and do not describe the potential for neural differentiation, slowing translation to the clinic. Therefore, we aimed to culture and characterize NCSCs from the human dental pulp in vitro and evaluate their ability to differentiate into neurons; we also investigated the effectiveness of the addition of BMP4 to enhance this potential. Cultures were established from a varied cohort of patient samples and grown, as monolayers, in serum, serum‐free, and also under sphere‐aggregation conditions to induce and identify a NCSC phenotype. hDPC cultures were characterized by immunocytochemistry and reverse transcription quantitative polymerase chain reaction. Monolayer cultures expressed stem cell, neural progenitor and neural crest‐related markers. Culturing hDPCs as neurospheres (hDPC‐NCSCs) resulted in an increased expression of neural crest‐related genes, while the addition of BMP4 appeared to produce better NCSC characteristics and neural differentiation. The neural‐like phenotype was evidenced by the expression of TUJ1, peripherin, NFH, TAU, SYN1, and GAP43. Our results describe the establishment of hDPC cultures from a large variety of patients in serum‐free medium, as NCSC that differentiate into neural‐like cells, as well as an important effect of BMP4 in enhancing the neural crest phenotype and differentiation of hDPCs.

## Graphical abstract

Human dental pulp tissue collected from a varied cohort of samples was used to directly establish monolayer cultures in defined serum‐free conditions. In monolayer, the cultures maintained an mesenchymal stem cell (MSC) phenotype regardless of the initial serum‐free conditions. A Neural crest‐derived stem cell phenotype was favored by adding BMP4 before or after transferring the cells to a 3D culture.

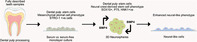

**Significance statement**

This work details the acquisition of cells from human dental pulp and describes their establishment and growth in preclinically relevant, serum‐free culture conditions, in comparison to standard serum‐rich cultures. In contrast with the current literature, this article described in greater detail the nature and condition of the samples used from a large group of patients, allowing for a greater understanding in relation to clinical translation. Results provide evidence of neural crest‐related markers in all growing conditions, and the improvement of a neural crest‐like phenotype by growing human dental pulp cells (hDPCs) in 3D neurospheres. Importantly, this article provided evidence that hDPCs that are characterized as neural crest‐derived stem cells can differentiate into neural‐like cells, as well as describing an important novel effect of BMP4 on hDPC neural crest‐like phenotype.

## 1 INTRODUCTION

Oral‐derived stem cells (OSCs) have been generally characterized in vitro as mesenchymal stem cells (MSCs). These OSCs include the widely studied dental pulp cells as well as stem cell populations derived from other novel oral sources such as periapical Cyst‐MSCs (PCy‐MSCs). OSCs are regarded as good candidates for autologous cell therapies in regenerative medicine due to their multipotent differentiation, proliferative capacity, and accessibility (as reviewed by Tatullo et al).^1^

However, a factor that can delay translation of OSCs into the clinic is that the isolation, culture, and characterization of these stem cell populations have been commonly performed in medium containing fetal bovine serum (FBS; recently reviewed by Luo et al^2^ and Rodas‐Junco et al^3^), which is inappropriate for most applications in man. Hence, there is a need for protocols to establish cell cultures in conditions that would be more appropriate for advancing their translational use.^4^, ^5^ Thus, it is important to develop methods that would allow the establishment of cultures in serum‐free and/or xeno‐free conditions that could comply with clinical‐grade manufacturing standards in the near future. At the same time, there is a need to describe in more detail the possibility of using these cells from samples obtained from a wide variety of patients that could realistically resemble common clinical scenarios.

Most dental‐related stem cell sources are believed to derive from the neural crest,^6^ and because of this there is an increasing interest in the study of their neural crest‐derived stem cell (NCSC) characteristics.^7^, ^8^, ^9^ In contrast to MSCs from non‐neural crest origin, these NCSCs could prove particularly useful to restore neural cell types due to their molecular resemblance to embryonic neural crest cells.^10^, ^11^

In this regard, there is little information about the identification and isolation of a human NCSC population specifically from human dental pulp cells (hDPCs) of adult, permanent teeth, and only a small number of key direct applications have been reported.^12^, ^13^, ^14^ Thus, it is highly relevant to further our understanding of NCSCs from hDPCs, and also to test their proposed potential in relevant models of nerve regeneration.

Therefore, we present here a detailed description of the establishment and characterization of hDPCs grown in serum‐free media in vitro, the characterization of their molecular NCSC signature, and the role of BMP4 in enhancing this signature. In addition, we present proof of concept that these NCSCs, generated under serum‐free conditions from hDPCs, can derive neural‐like cells, proposing them as candidates for neural regeneration treatments.

## 2 MATERIALS AND METHODS

### 2.1 Dental pulp collection

The study was conducted in accordance with ethical approval granted by the Leeds East Research Ethics Committee of the UK National Research Ethics Service (reference: 15/YH/0308; protocol: STH19019). Human dental pulp was isolated from samples collected with written informed consent from patients at the Charles Clifford Dental Hospital, Sheffield, UK. Details of the patients' age and the condition and position of the tooth are provided in Table 1. Immediately after extraction, the samples were transported in ice‐cold PBS with 1× Penicillin‐Streptomycin (1× P/S; 100 IU/mL‐100 μg/mL; GIBCO, Paisley, UK) to the laboratory for processing.

**TABLE 1:** Human dental pulp samples

Sample ID	Patient age	Sex	Remarks (tooth position)	Medium	Successful
1	61	F	Periodontal disease	FBS	No
2	52	F	Caries (5)	FBS	No
3	19	F	Non‐carious, unerupted (8)	FBS	Yes
4	23	F	Non‐carious (8)	FBS	Yes
5	22	F	Non‐carious (8)	FBS	Yes
6	20	F	Fragmented (8)	FBS	Yes
7	27	F	Minimal Caries (8)	FBS	Yes
9	28	F	Fragmented (8)	N/A	N/A
16	16	F	Non‐carious (8)	FBS	Yes
16	16	F	Non‐carious (8)	OSCFM	Yes
17	22	M	Fragmented (8)	FBS	Yes
18	30	F	Non‐carious (8)	FBS	Yes
19	27	F	Non‐carious (8) (facial pain)	FBS	Yes
19	27	F	Non‐carious (8) (facial pain)	OSCFM	Yes
20	27	F	Non‐carious (8) (facial pain)	FBS	Yes
20	27	F	Non‐carious (8) (facial pain)	OSCFM	Yes
21	21	M	Non‐carious (8)	OSCFM	Yes
22	24	F	Non‐carious (8)	FBS	Yes
23	24	F	Minimal caries (8)	FBS	Yes
24	26	F	Non‐carious (8)	OSCFM	Yes
25	33	F	Non‐carious (8)	BMP4	Yes
26	15	F	Minimal caries (7)	OSCFM	No
27	20	F	N/S (8)	OSCFM	No
28	36	F	Minimal caries (8)	N/S	No
29	36	F	Non‐carious (8)	BMP4	Yes
32	35	M	Fragmented (?)	OSCFM	No
34	24	F	Non‐carious (8)	BMP4	No
34	24	F	Non‐carious (8)	OSCFM	Yes
37	38	F	N/S‐Mobile (8)	BMP4	No
38	38	F	Periodontal disease (?)	OSCFM	No
39	17	M	Non‐carious (3)	BMP4	No
39	17	M	Non‐carious (3)	OSCFM	Yes
40	17	M	Non‐carious (3)	BMP4	Yes
41	17	M	Non‐carious (3)	BMP4	Yes
42	20	F	Non‐carious (8)	BMP4	No
43	22	F	Non‐carious (8)	BMP4	Yes
44	29	M	Non‐carious (8)	FBS	Yes
45	29	M	N/S (8)	OSCFM	Yes
46	N/S	N/S	N/S	OSCFM	Yes
47	27	M	Minimal caries (8)	BMP4	No
48	27	M	Minimal caries (6)	FBS	Yes
49	39	M	Fragmented (8)	OSCFM	No
50	28	M	Minimal caries (6)	BMP4	No
51	31	M	Fragmented (8)	FBS	No
52	45	M	Fragmented (8)	BMP4	No
53	21	F	Non‐carious (8)	BMP4	No
53	21	F	Non‐carious (8)	OSCFM	Yes
54	17	F	Non‐carious (2)	FBS	No
54	17	F	Non‐carious (2)	OSCFM	No
55	17	F	Non‐carious (5)	BMP4	No
56	37	F	N/S	BMP4	No
56	37	F	N/S	OSCFM	No
57	37	F	Minimal caries (?)	FBS	Yes
57	37	F	Minimal caries (?)	OSCFM	Yes
58	37	F	Minimal caries (?)	BMP4	No
58	37	F	Minimal caries (?)	FBS	Yes
59	N/S	N/S	Non‐carious (?)	FBS	Yes
60	47	M	Minimal caries (8)	BMP4	No

*Note*: In some cases, the same sample digest was split and cultured in two different media (samples 19, 20, 54, 56, 57, 58). Numbers in parentheses indicate tooth position.

Abbreviations: BMP4, bone morphogenic protein; F, female; FBS, fetal bovine serum; hDPC, human dental pulp cells; M, male; N/A, not applicable; N/S, not specified; (?), not specified; OSCFM, otic stem cell full medium.

### 2.2 Dental pulp cell culture establishment

Dental pulp was extracted by making a longitudinal groove on the tooth with an electric drill cooled under running water and sectioning the tooth with a mallet and osteotome to expose the pulp. The dental pulp was removed, cut into small pieces and digested with collagenase IV (3 mg/mL; GIBCO) for 60 minutes, aided by pipetting every 30 minutes. The dissociated tissue was passed through a 100 μm strainer, washed with PBS, and centrifuged for 5 minutes at 400*g*. The pellet was resuspended directly into either: (a) standard medium: DMEM, 1× P/S, 1× Glutamax (GIBCO), 10‐4 Ascorbic Acid (Sigma, Dorset, UK), 20% FBS (GIBCO); (b) otic stem cell full medium (OSCFM): DFNB (DMEM:F12 (GIBCO), 1% N2 (GIBCO), 2% B27 (GIBCO), 1× P/S (SIGMA), 20 ng/mL bFGF (R&D Systems, Abingdon, UK), 50 ng/mL IGF (R&D Systems), 20 ng/mL EGF (R&D Systems); or (c) bone morphogenic protein 4 (BMP4) medium: OSCFM supplemented with 10 ng/mL BMP4 (R&D Systems). OSCFM and BMP4 cultured cells were grown on laminin‐coated six well plates (2.5 μg/cm^2^; Cultrex, Gaithersburg, MD). The time between sample collection and their final deposition in culture conditions was less than 4 hours. The cultures remained undisturbed for the first 4 days. From day 4, half a volume of the respective media was replaced. From day 12 onward, all medium was replaced every 2 to 3 days.

Primary cultures were passaged at week 4, and cultures able to proliferate and survive at least four passages were described as successful cultures (“Yes” or “No”; Table 1). Following the initial passage, cultures were passaged every week at 5000 to 10 000 cells/cm^2^ using 1:10 Trypsin EDTA (TE; Sigma) for FBS cultures and 1:80 TE for OSCFM and BMP4 cultures.

### 2.3 Calculation of cumulative cell doublings

The number of living cells was obtained by trypan blue staining, and cell number was counted using the automated cell counter TC20 (Bio‐rad, Hertfordshire, UK; particle size range 9‐21 μm). cumulative cell doubling (CCD) of living cells was calculated using the following formula:

$$\mathrm{CCD}=3.32\;\left(\log\;\mathrm{UCY}\;-\;\log\;I\right)\;+\;X$$

where UCY is the final cell count, I is the initial cell number seeded, and X the previous cell doubling number (American Type Culture Collection).

### 2.4 Neurosphere formation

hDPC cultures were detached with the corresponding Trypsin EDTA treatment (as described above) and resuspended in sphere medium: DFNB, 1× P/S (Sigma), 20 ng/mL bFGF (R&D Systems), 20 ng/mL EGF (R&D Systems) at 10 000 cells per 100 μL in ultra‐low attachment culture plates (Costar, UK). Neurospheres were fed by adding 50 μL of the medium at day 3 and 6. The number of individual spheres and their diameter was measured on day 4. Neurospheres were harvested or used in downstream applications from days 6 to 8. Where indicated, 10 ng/mL BMP4 (R&D Systems) was supplemented to the sphere medium.

### 2.5 Neural differentiation of hDPC‐NCSCs

Neurospheres were induced as described by Gervois et al^15^ with modifications. At day 6 to 8, neurospheres were transferred to Poly‐ornithine (0.01%; Sigma)/Laminin (2.5 μg/cm^2^; Cultrex) coated plates in neuralizing medium consisting of DFNB, 30 ng/mL NT3 (Prepotech, London, UK) and 1 mM dbcAMP (Tocris, Abingdon, UK). The medium was replaced every 2 to 3 days for up to 2 weeks.

### 2.6 Transference of hDPC‐FBS cells to OSCFM and BMP4 conditions

hDPC‐FBS cells were transferred to the conditions described for OSCFM and BMP4 cultures for 7 days. Cells maintained in FBS conditions were used as a control. After the 7 days of the transference, hDPC‐NCSC neurospheres were formed. hDPC‐neurospheres were then induced for neural differentiation as described above.

### 2.7 Neurosphere sectioning

After fixation (see below), hDPC neurospheres were subjected to a sucrose gradient of 7.5%, 15%, 22%, and 30% sucrose (12‐24 hours each; 4°C), and transferred to optimal cutting temperature (OCT) solution for 24 hours (4°C). The tissue was then fast‐frozen on a dry ice/methyl‐butane bath. Cryosectioning was then performed in a Bright OTF5000 cryostat in sections of 5 to 10 μm.

### 2.8 Fixing, immunocytochemistry, and imaging

Cells and spheres were fixed with 4% Methanol‐free PFA (Alfa Aestar, Lancashire, UK) at room temperature for 10 minutes. For immunocytochemistry, cultures were blocked for 1 hour with 5% normal donkey serum‐PBST (Sigma; Blocking solution). Primary antibodies were added and left overnight at 4°C. Secondary antibodies were added in blocking solution (as above) for 1 hour at room temperature together with DAPI nuclei counterstaining (1:100).

Antibodies and dilutions: RBT NFH (NF200; 1:100; Sigma, N4142), RBT PER (1:100; Millipore, Watford, UK, AB1530), MSE TUJ1 (1:100; Biolegend, London, UK, 801202), RBT TAU (1:100; Santa Cruz Biotechnology, Heidelberg, Germany, [H‐150] 5587), RBT SOX2 (1:100; Millipore, AB5603), MSE AP2a (1:100; Santa Cruz Biotechnology, SC12726), MSE NESTIN (1:100; [10c2], Santa Cruz Biotechnology, SC23927), MSE P75 supernatant (1:10; Centre for Stem Cell Biology, Sheffield, UK ME 20.4), MSE SLUG (1:100; Santa Cruz Biotechnology, [A‐7] SC166476), MSE SNAIL1 (1:100; Santa Cruz Biotechnology, [G‐7] SC271977), Goat SOX10 (1:75; Santa Cruz Biotechnology, SC17342), RBT SOX9 (1:100; Millipore, AB5535), MSE STRO‐1 (1:100; R&D Systems, MAB1038). DNKa‐MSE Alexa 488 (1:250; Invitrogen, Loughborough, UK, A21202), DNKa‐RBT‐Alexa 568 (1:250; Invitrogen, A10042), DNKa‐GOAT Alexa 568 (1:250; Invitrogen, A11057).

The EVOS cell imaging system and InCell Analyzer inverted microscopes were used for fluorescent and brightfield imaging. FIJI image software was used for image processing and analysis. Negative controls were calculated by omitting the primary antibody during the immunocytochemistry.

### 2.9 P75 and STRO‐1 positive cell population counting

Cultures positively stained for nerve growth factor receptor (P75) and STRO‐1 at passage 0 and between passage 2 and 4 were manually counted and the percentage of positive cells was calculated based on the total cell number defined by DAPI nuclei staining.

### 2.10 RNA extraction and cDNA synthesis

The Qiagen microkit (Qiagen, Manchester, UK) was used to extract RNA from the cell cultures according to the manufacturer's instructions and quantified using a Nanodrop spectrophotometer. For cDNA synthesis, at least 200 ng of RNA was used with the Superscript IV retro transcriptase (Thermofisher, Loughborough, UK) according to the manufacturer's instructions.

### 2.11 Direct RT‐qPCR preparation

hDPC‐FBS transferred into the other culture conditions (FBS, OSCFM, and BMP4) and induced to neural differentiation (refer to Figure 6D) were analyzed using a Cells to CT kit (Thermofisher) according to the manufacturer's instructions.

### 2.12 Quantitative polymerase chain reaction

Reverse transcription quantitative polymerase chain reaction (RT‐qPCR) was performed with Taqman probes using the Thermofisher QuantStudio 12 k Flex Real‐time PCR system. The reactions were prepared with at least 1 ng/μL of cDNA. The master‐mix, assays, and water volumes were used according to the concentrations specified by the manufacturer's instructions in a final volume of 7.5 μL per reaction in quadruplicate. The relative expression analysis was performed using the 2^ddCT^ method.

Taqman probes: RPLP0 (Hs00420895_gH), SOX2 (Hs00602736_s1), OCT4/POUF5F1 (Hs04260367_gH), NANOG (Hs02387400_g1), SOX10 (Hs00366918_m1), P75/NGFR (Hs00609976_m1), NESTIN (Hs04187831_g1), SNAIL1 (Hs00195591_m1), AP2a/TFAP2A (Hs01029413_m1), SYN1 (Hs00199577_m1), NFH/NEFH (Hs00606024_m1), GAP43 (Hs00967138_m1).

### 2.13 Statistical analysis

One‐way ANOVA followed by a Sidak's or Tukey test for pairwise comparison was made to determine differences. A linear regression analysis was used to determine slope differences. The tests were run in GraphPad Prism version 8.3.0 (GraphPad Software, San Diego, California).

## 3 RESULTS

### 3.1 hDPCs can be established in serum‐free conditions from a variety of samples

With the purpose of establishing in vitro hDPC cultures, 48 teeth (molars, premolars, canines, and incisors) were collected with written informed consent from patients at the Charles Clifford Dental Hospital, Sheffield, UK. Patient data collected comprised age, sex, and reason for tooth extraction as described by the clinical staff (Table 1). The age of the patients ranged from 15 to 61 years, with a mean of 27 (±8.9) years, a median of 26 years and a mode of 27 years. Carious teeth were included only if the caries was considered by the clinical staff to be minimal. Samples that were fragmented during tooth removal were omitted, as were the samples from the two oldest patients with poor, overall oral health (severe periodontal disease, pulpitis; samples 1 and 2; Table 1).

**FIGURE 1:**
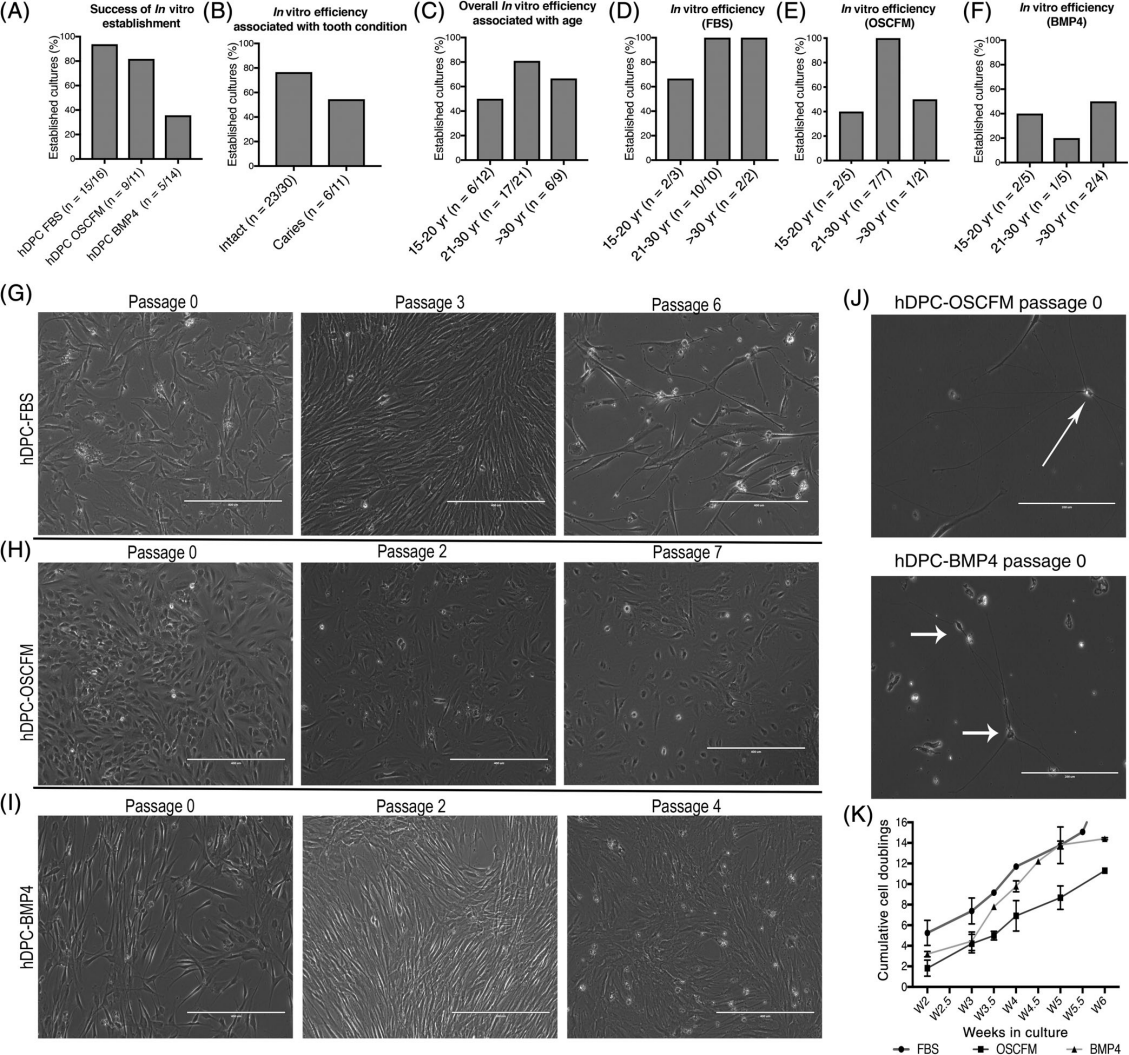
hDPC culture establishment. The percentage success for individual samples to grow in vitro and be cultured for at least four passages was calculated under the following criteria: A, total samples collected for culture establishment; B, tooth condition; C, overall success associated with age; D, success associated with age in cultures grown in FBS medium; E, success associated with age in cultures grown in OSCFM medium; and F, success associated with age in cultures grown in BMP4 medium. Comparison of independent cultures grown in different conditions and changes in relation to their passage number: G, FBS cultures; H, OSCFM cultures; I, BMP4 cultures. J, hDPC‐OSCFM (upper) and hDPC‐BMP4 (lower) at passage 0 showing neural‐like cells not present in later passages (arrows). K, Cumulative cell doublings describing the proliferative ability of hDPC cultures able to be passaged for more than four passages in the different culture conditions. Overall slopes and elevation were statistically similar (*P* > .05). Scale bars: G‐I = 400 μm; J = 200 μm. BMP4, bone morphogenic protein 4; FBS, fetal bovine serum; hDPC, human dental pulp cells; hDPC‐FBS, hDPC grown in 20% FBS, hDPC‐OSCFM, hDPC grown in OSCFM; hDPC‐BMP4, hPDC grown in OSCFM supplemented with BMP4; OSCFM, otic stem cell full medium

To determine whether hDPC primary cultures could be established in serum‐free, neurogenic conditions from the beginning, freshly dissociated hDPCs were seeded in either OSCFM (a medium used previously to isolate otic progenitors from the fetal human cochlea^16^) or OSCFM supplemented with BMP4, and compared with seeding in standard 20% serum‐DMEM. The in vitro efficiency of each culture system was determined by recording the number of cultures that managed to proliferate and be passaged more than four times. The graphs shown in Figure 1A‐F represent the percentage of successful cultures. The highest success rate of surviving cultures was FBS (93%, n = 15/16), followed by OSCFM (81%, n = 9/11) and BMP4 (35%, n = 5/14; Figure 1A). To consider common situations in the clinical setting, we also included mildly carious teeth in our experiments. Overall, dental pulp culture establishment from non‐carious teeth had 76% efficiency (n = 23/30), while in carious teeth the efficiency dropped to 54% (n = 6/11; Figure 1B).

We also calculated the overall culture efficiency in relation to age, by grouping the samples in the following age groups: group 1: 15‐20 years (50%, n = 6/12), group 2: 21‐30 years (80%, n = 17/21), and group 3: >30 years (66%, n = 6/9; Figure 1C). However, consideration should also be made for each culture condition. FBS cultures were highly successful throughout, with 100% in group 2 (n = 10/10) and group 3 samples (n = 2/2), and the lowest in the youngest samples (n = 2/3), with 66% success (Figure 1D). OSCFM cultures were established with greater frequency from samples in the middle group (100%; n = 7/7), followed by the youngest with 40% (n = 2/5) and oldest with 50% (n = 1/2) success rate (Figure 1E). On the other hand, BMP4 cultures had the lowest efficiency rate overall with the youngest group having 40% success rate (n = 2/5), the middle group 20% (n = 1/5), and the oldest aged patient group 50% (n = 2/4; Figure 1F).

Culture passaging showed an effect on cell morphology and density regardless of the initial culture condition. Distinct differences in cell morphology were evident between our culture conditions: hDPC‐FBS (Figure 1G), hDPC‐OSCFM (Figure 1H), and hDPC‐BMP4 (Figure 1I). Notably, neural‐shaped cells were evident at passage 0 in the neurogenic conditions OSCFM and BMP4 but disappeared soon after passaging (Figure 1J, arrows). Once established, we calculated CCDs. After comparison of the growth curves, the analysis showed no differences between the growth of hDPC in the different culture conditions (Figure 1K).

### 3.2 hDPC cultures express neural crest‐related markers

**FIGURE 2:**
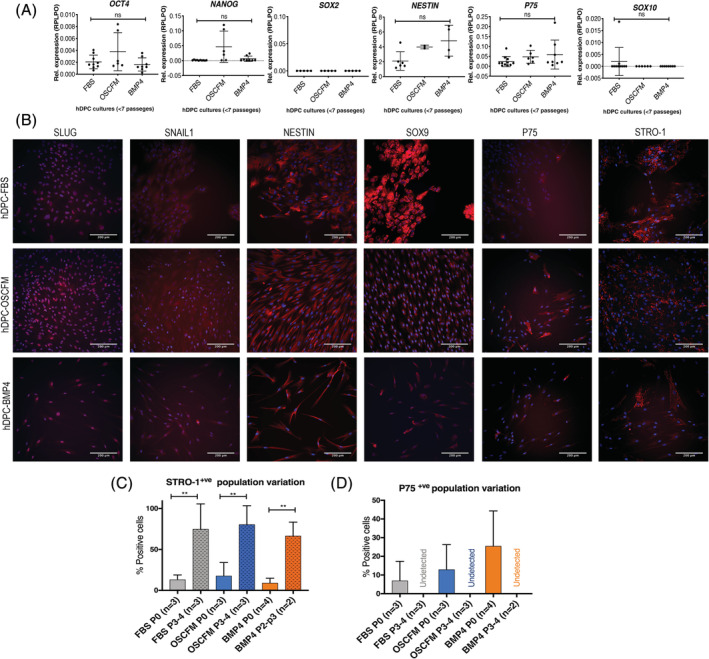
Human dental pulp cells (hDPCs) characterization. A, Basal relative expression of pluripotent and neural crest markers in hDPC cultures established directly into FBS, OSCFM, or BMP4 medium at passages 0 to 7. Each data point represents independent cultures normalized to the expression of the embryonic stem cell line H14 S9 established as 1 (not illustrated). B, Representative images of the immunocytochemistry of neural crest‐related markers and STRO‐1 expressed in at least three independent cultures of hDPCs at passages 0 to 7. P75 panels represent cultures at passage 0. Scale bar = 200 μm. C) Quantification of STRO‐1^+ve^ cells at different passage number in the three different conditions in independent experiments. D, Quantification of P75^+ve^ cells at different passage number in the three different conditions in independent experiments. **P < .01; one‐way ANOVA followed by Sidak's pairwise comparison test. n is the number of independent cultures. ANOVA, analysis of variance; BMP4, bone morphogenic protein 4; FBS, fetal bovine serum; OSCFM, otic stem cell full medium

We aimed to define the core molecular signature of hDPCs when cells were grown in two‐dimensional, monolayer cultures in the three different conditions. A relative expression analysis by RT‐qPCR was performed to identify the expression of pluripotent stem cell and neural crest‐related genes. When compared to a human pluripotent embryonic cell line, low relative expression levels of *OCT4*, *NANOG*, and *P75* were detected in all our culture conditions. *NESTIN* was expressed in all culture conditions with a higher relative expression. Notably, *SOX2* and S*OX10* expression were undetected. None of the evaluated markers presented a statistically significant difference among FBS, OSCFM, or BMP4 conditions (Figure 2A).

Using immunocytochemistry for common neural crest markers, the presence of SLUG, SNAIL1, NESTIN, SOX9, and P75 revealed a characteristic NCSC signature in monolayer cultures (Figure 2B). However, markers such as SOX10 (not shown) and P75 were not recognized or only detected at an earlier passage number, respectively. In particular, P75 and STRO‐1 were present in some cells within the cultures, evidencing culture heterogeneity (Figure 2B). We further quantified culture heterogeneity by calculating the percentage of STRO‐1^+ve^ and P75^+ve^ cells at passage 0 and comparing them to a later passage. The STRO‐1^+ve^ population increased significantly after culture passaging in all media conditions, while P75 was only detectable at passage 0, suggesting subpopulations change dynamically during passages in culture (Figure 2C,D).

### 3.3 NCSC markers are enhanced when hDPCs are grown as spheres

Due to the evident changes in sub‐populations occurring during expansion as monolayers (Figure 2C,D), it was hypothesized that culturing hDPCs as 3D aggregates could drive them into a more stable, NCSC phenotype (hDPC‐NCSC). Cells initially expanded as a monolayer were passaged and forced to aggregate into neurospheres for characterization.

**FIGURE 3:**
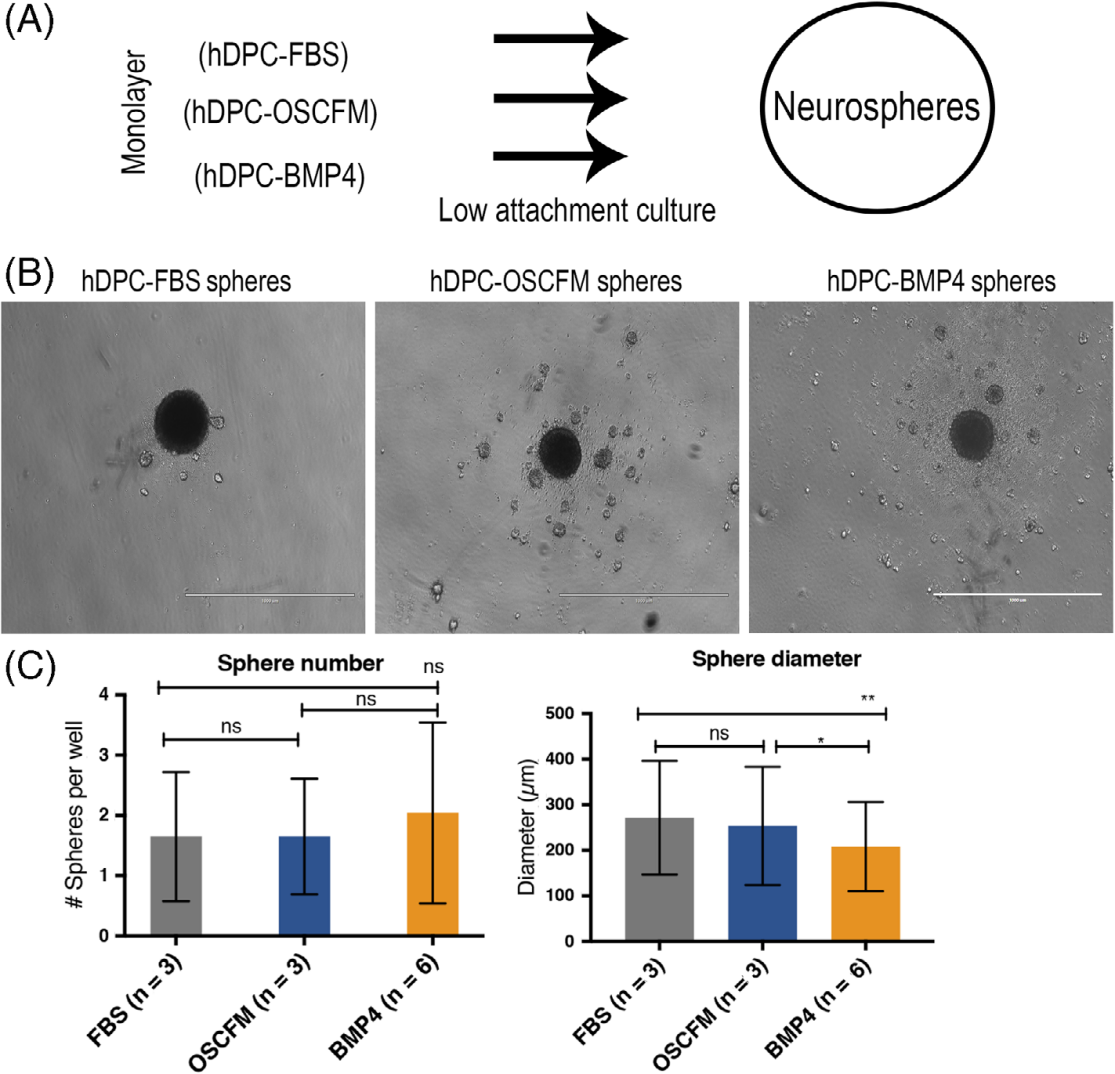
NCSC neurospheres formation. A, The workflow of neurosphere formation from all culture conditions. B, hDPCs from FBS, OSCFM, and BMP4 conditions cultured in low‐attachment wells. C, Sphere number and diameter per well were calculated from at least 10 wells per independent culture (n = 3 or 6). Neurospheres were counted if diameter >100 μm. *P < .05, **P < .01; one‐way ANOVA followed by Tukey's test for pairwise comparison. n is the number of independent cultures. ANOVA, analysis of variance; BMP4, bone morphogenic protein 4; FBS, fetal bovine serum; hDPC, human dental pulp cells; NCSC, neural crest‐derived stem cells; OSCFM, otic stem cell full medium

An equal number of cells from each initial culture condition were transferred to low attachment culture plates, and sphere formation (as number of spheres per well and diameter) was assessed after 4 days in culture (Figure 3A,B). Overall, no significant difference was observed in sphere number among our culture conditions, and a statistically significant smaller diameter was observed in spheres derived from BMP4 cultures (Figure 3C).

**FIGURE 4:**
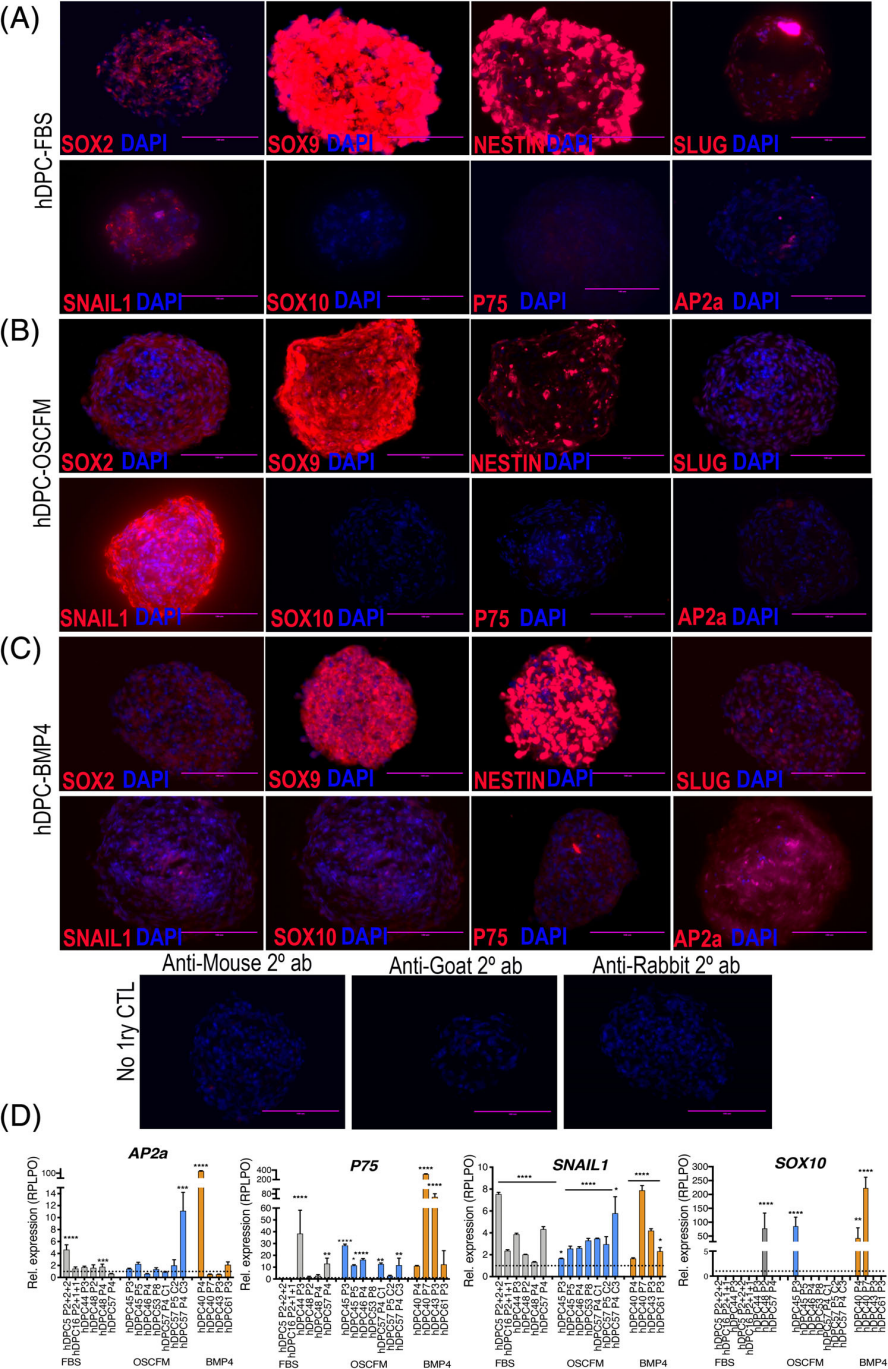
NCSC neurospheres characterization. Immunolabeling for neural crest‐related markers in hDPC‐neurospheres derived from A, hDPC‐FBS cultures; B, hDPC‐OSCFM cultures; and C, hDPC‐BMP4 cultures. “No 1ry CTL”: controls with no primary antibody, showing a representative view of neurospheres labeled with the secondary antibodies only. D, Neurospheres from all conditions (FBS‐grey, OSCFM‐blue and BMP4‐orange) were analyzed by RT‐qPCR for the expression of NCSC markers: SNAIL1, P75, SOX10, and AP2a. Each bar represents an independent neurosphere culture that was normalized and compared to their own monolayer counterpart (dotted line = 1). Error bars represent technical replicates of each independent sample. Scale bars = 100 μm (A‐C). A‐C, Representative pictures of multiple spheres from at least three independent cultures. A one‐way ANOVA followed by a Sidak's multiple comparison test was made to determine differences. *P < .05, **P < .01, ***P < .001, ****P < .0001. 1ry, primary; BMP4, bone morphogenic protein 4; CTL, control; FBS, fetal bovine serum; hDPC, human dental pulp cells; hDPC‐FBS, hDPC grown in 20% FBS, hDPC‐OSCFM, hDPC grown in OSCFM; hDPC‐BMP4, hPDC grown in OSCFM supplemented with BMP4; NCSC, neural crest‐derived stem cells; OSCFM, otic stem cell full medium

Neurospheres were then analyzed by immunocytochemistry to identify neural crest‐related markers. hDPC‐FBS‐ (Figure 4A), hDPC‐OSCFM‐ (Figure 4B), and hDC‐BMP4‐ (Figure 4C) neurospheres labeled positively for SOX2, SOX9, NESTIN, SLUG, and SNAIL1. However, the markers AP2a, SOX10, and P75 were predominantly present in hDPC‐BMP4 neurospheres (Figure 4C), while were almost undetected in the hDPC‐FBS and hDPC‐OSCFM neurospheres (Figure 4A,B). Interestingly, SOX2 was expressed by neurospheres from all culture conditions, while appeared undetected by RT‐qPCR in the earlier monolayer cultures.

To support the observation made by immunocytochemistry, we also studied the expression of *SNAIL1*, *P75*, *SOX10*, and *AP2a* quantitatively by RT‐qPCR. To do this, we analyzed the relative expression levels of each neurosphere sample in comparison to their own monolayer counterpart. Overall, *P75* and *SNAIL1* expression was significantly higher in the majority of independent neurospheres from all conditions (Figure 4D), whereas *SOX10* and *AP2a* were upregulated only in a few samples grown as neurospheres (Figure 4D). Overall, immunocytochemistry and RT‐qPCR data suggested that hDPC‐derived spheres presented a more robust upregulation of neural crest‐related markers in relation to their monolayer counterparts, thus supporting a NCSCs phenotype (hDPC‐NCSCs). The sample‐to‐sample variation that can be observed from the RT‐qPCR data evidenced the natural variability and heterogeneity of independent donors and passage number.

### 3.4 BMP4 can enhance the NCSC phenotype from hDPC‐FBS cultures

**FIGURE 5:**
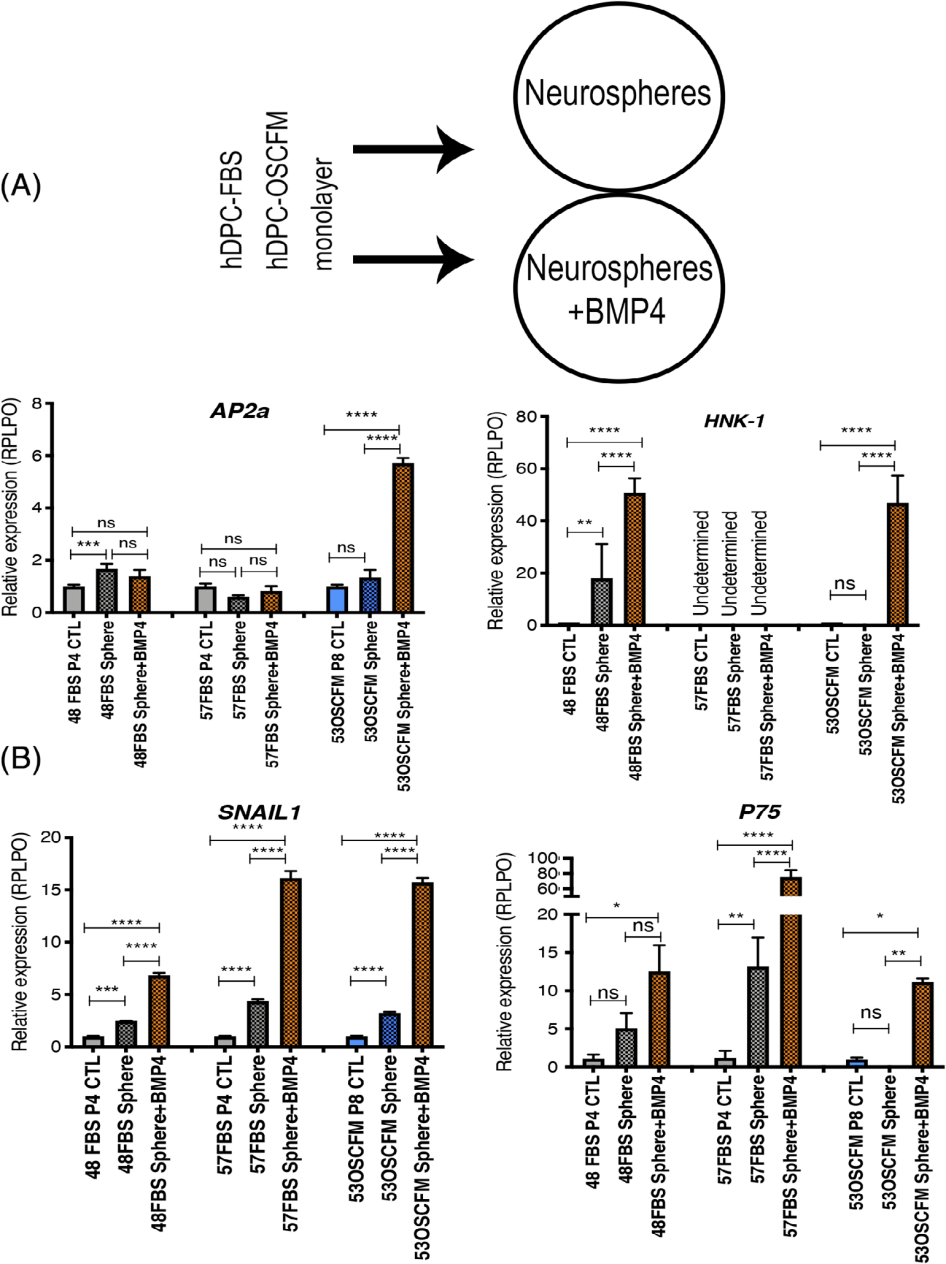
NCSC neurospheres characterization and BMP4 effect in NCSC phenotype. A, The workflow of neurosphere formation from independent hDPC‐FBS and hDPC‐OSCFM cultures with and without the addition of BMP4. B, Neurospheres from independent hDPC‐FBS and hDPC‐OSCFM cultures were supplemented with 10 ng/mL BMP4 during sphere formation phase. The expression of neural‐crest related markers: *Ap2a*, *SNAIL1*, and *P75* in the neurospheres was analyzed by RT‐qPCR and compared to the monolayer parental line. Error bars represent technical replicates of each independent sample. A one‐way ANOVA followed by a Tukey's test for pairwise comparison was made to determine differences. *P < .05, **P < .01, ***P < .001, ****P < .0001. BMP4, bone morphogenic protein 4; FBS, fetal bovine serum; hDPC, human dental pulp cells; hDPC‐FBS, hDPC grown in 20% FBS, hDPC‐OSCFM, hDPC grown in OSCFM; hDPC‐BMP4, hPDC grown in OSCFM supplemented with BMP4; NCSC, neural crest‐derived stem cells; OSCFM, otic stem cell full medium

As BMP4 has been shown to drive NCSC identity,^17^ we evaluated whether BMP4 could affect the NCSC phenotype if used during sphere formation rather than during culture establishment (Figure 5A). Two hDPC‐FBS cultures and one hDPC‐OSCFM culture (also included in the previous analysis; Figure 5B) were supplemented with BMP4 during sphere aggregation. RT‐qPCR was used to determine any particular change in the NCSC phenotype and compared to the neurosphere conditions described above, as well as the cells grown as monolayers (baseline control, CTL). As seen before, *AP2a* showed the most limited upregulation in which only the 53OSCFM neurospheres showed a positive regulation when supplemented with BMP4 (Figure 5B). *HNK‐1* presented a significant upregulation in two of the three independent samples analyzed. Interestingly, the three hDPC cultures selected for this experiment showed a significant upregulation of *SNAIL1* and *P75* expression in comparison to both CTL and neurospheres without BMP4 (except *P75*‐ 48FBS Sphere vs 48FBS sphere+BMP4, where the difference showed a trend but was not large enough to be significant). All the evidence together suggests that the addition of BMP4 to the neurospheres can induce even higher relative expression levels of important neural crest‐related genes.

### 3.5 hDPCs‐NCSCs differentiate in vitro into neural‐like cells

**FIGURE 6:**
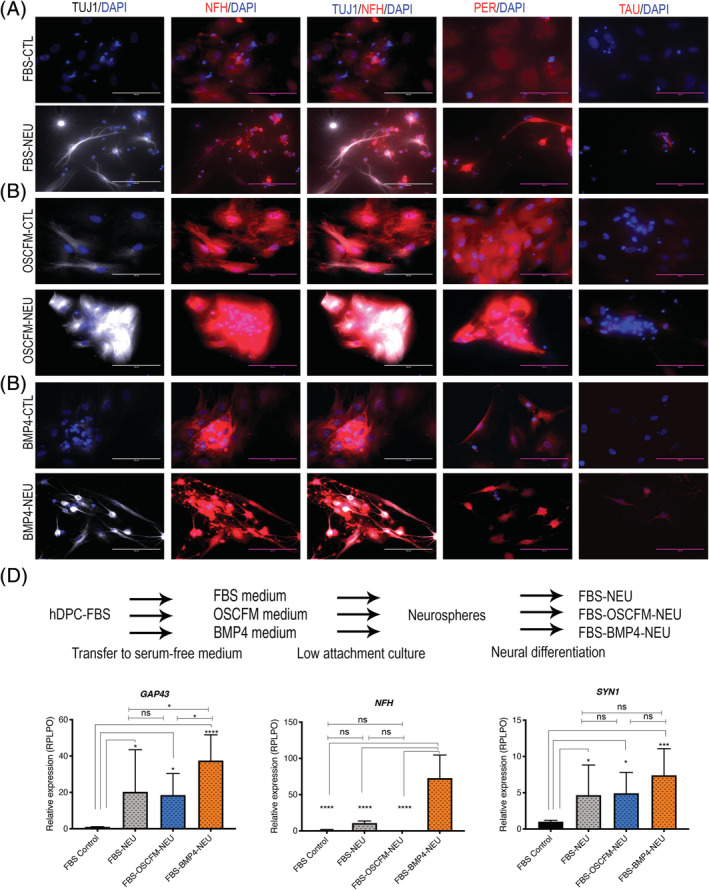
hDPC‐NCSCs neural differentiation. Neural differentiation of NCSCs. Representative pictures from at least three independent cultures from A, hDPC‐FBS; B, hDPC‐OSCFM; and C, hDPC‐BMP4 cultures. The expression of neural‐related markers TUJ1, NFH, peripherin (PER), and TAU was analyzed by immunocytochemistry in NCSC induced to neural differentiation (NEU) and compared to their undifferentiated control (CTL). Neural morphology and an apparently stronger signal were observed in hDPC‐NEU cells compared to controls (A‐C). Only BMP4‐NEU showed expression of the four analyzed markers (C). Scale bar = 100 μm (A‐C). D, hDPC‐FBS cells were transferred to OSCFM and BMP4 culture conditions before neurosphere formation and were subject to neural differentiation. The gene expression of neural related markers *GAP43*, *NFH*, and *SYN1* were upregulated mainly in the neural differentiated cells from hDPC‐FBS transferred to the BMP4 condition (FBS‐BMP4‐NEU). One‐way ANOVA followed by Tukey's test for pairwise comparison. *P < .05, ****P < .0001. BMP4, bone morphogenic protein 4; CTL, control; FBS, fetal bovine serum; hDPC, human dental pulp cells; hDPC‐FBS, hDPC grown in 20% FBS, hDPC‐OSCFM, hDPC grown in OSCFM; hDPC‐BMP4, hPDC grown in OSCFM supplemented with BMP4; NCSC, neural crest‐derived stem cells; OSCFM, otic stem cell full medium

hDPC‐NCSCs (neurospheres) derived from every initial experimental condition (Figure 3A) were induced to differentiate into neurons by culturing them for up to 2 weeks with dbcAMP and NT3. As control, equivalent hDPC‐NCSCs were transferred to the neural differentiation media, without dbcAMP and NT3. Compared to the undifferentiated controls, many hDPCs‐NCSCs‐derived cells presented a neuronal‐like morphology extending projections and a qualitatively higher intensity of the neuronal markers TUJ1 and peripherin (PER; Figure 6A‐C). NFH was also expressed in the neural differentiated group colocalizing with TUJ1 in cultures from hDPC‐OSCFM and hDPC‐BMP4 (Figure 6B,C), but not from hDPC‐FBS cultures (Figure 6A). Furthermore, TAU expression was only detected in neuronal differentiated cells from hDPC‐BMP4 cultures (Figure 6C). Notably, cells with non‐neuronal morphology were frequently visible in neural induced cultures; additionally, cells were in some cases unable to grow out from the neurospheres or were nonviable (not shown).

### 3.6 Neural differentiation from hDPC‐FBS cultures can be enhanced when transferred to OSCFM and BMP4 culture conditions

To further explore the ability of BMP4 to support neurogenic cultures, hDPC‐FBS cells were transferred to OSCFM and BMP4 conditions for 7 days and then induced to neurosphere formation and neural differentiation (Figure 6D). RT‐qPCR was used to analyze the neural‐related genes *GAP43*, *NFH*, and *SYN1* after neural differentiation (Figure 6D). As a control, we used the parental hDPC‐FBS monolayer culture (Figure 6D). hDPC‐FBS cells transferred to OSCFM before neuralization (FBS‐OSCFM‐NEU) presented a similar behavior in comparison to neuralized cells from the initial FBS conditions (FBS‐NEU), as judged by the expression levels of *GAP43* and *SYN1*. Interestingly, *GAP43* and *NFH* were significantly overexpressed in the hDPC‐FBS cells transferred to the BMP4 conditions before neural differentiation (FBS‐BMP4‐NEU), in comparison to the rest of the experimental groups. Furthermore, FBS‐BMP4‐NEU cells also presented a higher significant upregulation of *SYN1* when compared to the control group (Figure 6D).

## 4 DISCUSSION

In the present work, we have described the establishment of dental pulp cultures from a wide variety of tooth sample conditions and ages. The range of samples presented allowed us to extract critical data in terms of efficiency, patient age, and sample condition, which should be considered for the translational research of hDPCs, and which are not commonly reported from such a varied sample cohort as this one. One of the most complete reports previously available, described a set of 40 samples from patients aged 18 to 30 years, showing 78% success rate to grow in vitro (but under standard FBS conditions),^18^ which is similar to the overall efficiency found in the present work. We have accounted for the presence of caries in our samples, and have shown that it is possible to extract viable cells of similar identity from affected teeth, although at a reduced efficiency (Table 1).

We have shown that hDPC can be established in OSCFM directly from the primary tissue with an efficiency of 81%, providing evidence of the suitability of this medium to be used for the establishment and expansion of hDPCs in culture. On the other hand, successfully established hDPC‐BMP4 cultures behaved similarly to hDPCs in FBS and OSCFM media, as judged by marker expression and CCDs. Nevertheless, the efficiency of establishing cultures in BMP4 was lower, with only a third of the attempts being successful. The need to use serum‐free and xeno‐free conditions for dental pulp cell cultures has been recognized, and several authors have described the growth of hDPC in defined media.^4^, ^5^, ^14^, ^19^, ^20^, ^21^ However, most of the abovementioned reports included serum in their initial culture establishment, jeopardizing the use of those cultures for translational applications including autologous cell therapies.^4^, ^5^, ^14^, ^19^, ^20^, ^21^, ^22^

We characterized hDPCs grown under OSCFM and BMP4 conditions by RT‐qPCR, and compared them to those derived in the presence of serum. We explored whether markers of pluripotency such as *SOX2*, *NANOG*, and *OCT4* were expressed by our cultures, since these have been identified before in hDPCs and other oral‐derived NCSCs, fueling the claim that these populations could be pluripotent.^23^, ^24^, ^25^, ^26^ Nevertheless, most of these reports have not used sensitive, quantitative technologies (eg, Taqman) to analyze the cultures and/or compared them to a truly pluripotent stem cell line, as we have done. Our results suggest that, although present, the levels of the pluripotent stem cell markers *OCT4* and *NANOG*, together with the lack of *SOX2* expression, were not sufficient to suggest a pluripotent state of hDPCs in any of our conditions. Regarding the NCSC phenotype, we observed the expression of a large panel of relevant neural crest‐related markers including SLUG, SNAIL1, SOX9, and NESTIN in our three initial, isolating culture conditions by immunocytochemistry as well as NESTIN by RT‐qPCR. Thus, we provide evidence of neural crest‐like phenotypes already in our monolayer cultures. However, the percentages of P75 and STRO‐1 positive cells varied with passage number. The P75 marker has been proposed as a neural crest marker for hDPC,^27^ while STRO‐1 has been widely studied as a mesenchymal stromal cell marker in hDPCs.^28^ Therefore, the decrease in cells positive for P75 coupled with the increase of STRO‐1 suggests that the monolayer culture conditions were supportive of a MSC phenotype rather than of a NCSC phenotype. The latter is further supported by the low relative expression levels of *P75* and that only two hDPC‐FBS cultures were expressing *SOX10* by RT‐qPCR in monolayer conditions. The variable behavior observed in all our cultures illustrates the heterogeneous nature of hDPCs when grown as a monolayer, regardless of the serum or growth factors used to supplement the initial cultures. Our findings somehow differ from the study by Al‐Zer et al^12^ that established hDPC cultures in a serum‐free neurogenic medium and found P75 and SOX10 expression by immunocytochemistry. The discrepancies could be attributed to the different protocol for culture establishment (explant outgrowth) and/or the coating matrix on the plate (ie, the use of neuregulin‐β1 and fibronectin).

Our findings led us to hypothesize that heterogeneity in our hDPC cultures could lead to inefficacy in differentiation and that an intermediate population may be required for neural maturation, as shown by observations from the literature on hDPC‐neural differentiation.^14^, ^29^, ^30^, ^31^ Together with increasing reports obtaining NCSCs from other dental‐derived stem cells,^7^, ^8^, ^32^, ^33^ it is suggested that a neural crest or neurogenic population needs to be induced or enriched. In this regard, sphere aggregation has been used to enrich for a neural crest‐like phenotype in other tissues.^15^, ^32^, ^34^ Recent reports have also supported the advantages of growing hDPC as sphere aggregates, including multilineage differentiation and neural crest‐derived phenotype. Nevertheless, those reports derive from cultures originally established in serum‐rich conditions, which could contain neural inhibitory factors.^35^, ^36^ Thus, we aimed to grow and characterize hDPC grown as neurospheres as a method to obtain a neural crest‐derived stem cell phenotype from all our conditions tested. Our sphere aggregates showed a significant upregulation of *SNAIL1* and *P75* mRNA levels, the latter particularly in OSCFM and BMP4 groups. An enhanced NCSC phenotype on BMP4‐derived spheres can be further supported by the expression of SOX2, P75, and SOX10, in contrast to the monolayer cells in which appeared undetected in later passaged cultures by immunocytochemistry and RT‐qPCR. Similarly, cultures from adult palatum, periodontal ligament, dental pulp, or hair follicles grown in defined conditions have been shown to express the neural crest markers Sox10, Sox9, Snail1, Slug, P75, and Ap2a.^14^, ^34^, ^37^, ^38^ Our data represent a broader NCSC characterization from adult human dental pulp compared to other authors,^12^, ^13^, ^14^ and validation that NCSCs can be captured, using 3D cultures, from hDPCs. A variable expression of NCSC markers was noted, however, even within the same conditions. This could be a result of the heterogeneity of the cultures and importantly, of patient‐to‐patient variation, as can be implied from previous reports.^6^, ^39^, ^40^ Such variations must be considered for future clinical applications.

The addition of BMP4 to the initial growing conditions generally increased the expression of important NCSC markers such as P75 and SOX10 in hDPC‐BMP4 neurospheres, as observed by immunocytochemistry and RT‐qPCR. Additionally, in vitro differentiation into neural‐like cells was qualitatively better from BMP4‐containing cultures, with cells developing neurites and expressing markers of maturing neurons such as NFH, TUJ1, peripherin (PER), TAU, GAP43, and SYN1. However, neuralization into more mature phenotypes would need to be improved further. Cultures from all conditions had undifferentiated, non‐neural cells and in general, cells failed to migrate out of the spheres. We cannot rule out the possibility of obtaining a mature neural phenotype from all our hDPC culture conditions if left for a longer period, as has been described by Gervois et al.^15^ Other authors have encountered similar problems, when only a small fraction of cells were able to trigger action potentials using the same protocol.^41^ It is possible that the heterogeneity present in hDPCs‐NCSCs limits a full neurogenic potential.^35^

However, the observed hDPC‐BMP4 NCSC phenotype, as well as the improved changes observed during their neural differentiation in vitro, support the important role of BMP4 in sensory neuron and cranio‐facial neural crest induction.^17^, ^30^, ^42^, ^43^ To provide further evidence of the importance of BMP4 for the NCSC phenotype and neural differentiation, we also tested the effect of supplementing hDPC‐NCSCs (spheres) with BMP4 and, independently, transferring standard hDPC‐FBS cells to BMP4 culture medium conditions before neural differentiation. As a result, we observed (a) the enhanced neural crest phenotype observed in neurosphere formation supplemented with BMP4, (b) the better neuralization observed in the FBS‐BMP4‐NEU experiments, and (c) the apparent faster and pronounced changes observed during neural differentiation of the basal hDPC‐BMP4 in vitro, support its important role for the neural crest phenotype. Our results suggest an important role of BMP4 in the neural crest‐like phenotype of hDPCs.

Notably, the characterization of dental‐related human NCSCs, including those derived from gingival tissue, periodontal tissue, exfoliated deciduous teeth, and adult human dental pulp, has gained increasing interest for their potential use in neurodegenerative disorders. Nevertheless, most reports are limited to describing the molecular resemblance to neural crest cells, while their actual differentiation capacity is usually left untested, masking their actual translational potential for regenerative medicine.^7^, ^8^, ^9^, ^32^, ^33^, ^37^, ^44^

Additionally, it would be important to evaluate a combination of strategies to facilitate the application of oral‐derived NCSCs in regenerative medicine, such as their incorporation in advanced manufactured scaffolds or conduits for nerve repair and tissue engineering (eg, bone regeneration).^9^, ^45^, ^46^

Finally, the field of dental pulp stem cells and their applications would benefit from addressing specific diseases or targeted applications from preclinically relevant established cultures if we aim to advance the use of dental pulp cells in regenerative medicine.

## 5 CONCLUSION

We have provided a detailed description of the culture of hDPC from a wide variety of teeth, as well as a robust characterization of the culture conditions. We have also shown that cells can be directly established in serum‐free medium in contrast to the widely used serum‐rich standard conditions. This should prove highly advantageous in a preclinical setting. Also, hDPCs present a basal neural crest‐like phenotype that can be enhanced when induced to form neurospheres (hDPC‐NCSC). Furthermore, BMP4 has a significant role in enhancing the neural crest‐like phenotype in human dental pulp. It is worth noting, however, that there is a substantial level of heterogeneity and patient‐to‐patient variation that should be considered for future clinical applications.

Altogether, the present work details the culture and characterization of NCSCs from a broad cohort of human dental pulp samples established in preclinically relevant serum‐free conditions. Our results should facilitate the culturing of these cells under good manufacturing practice‐compliant processes and encourage their use in disease‐specific models to advance their application in regenerative medicine.

## CONFLICT OF INTEREST

Although not directly related to the work presented here, Marcelo N. Rivolta is the founder of Rinri Therapeutics. The other authors declared no potential conflicts of interest.

## AUTHOR CONTRIBUTIONS

O.S.‐C.: collection and assembly of data, data analysis and interpretation, manuscript writing, final approval of manuscript; F.B.: conception and design, provision of study material, data analysis and interpretation, manuscript writing, final approval of manuscript; M.N.R.: conception and design, financial support, provision of study material, data analysis and interpretation, manuscript writing, final approval of manuscript.

## Data Availability

The data that support the findings of this study are available from the corresponding author upon reasonable request.
